# Supplementary material for: Deterministic processes dominate soil microbial community assembly in subalpine coniferous forests on the Loess Plateau
Source: PeerJ. 2019 May 7;7:e6746. doi: 10.7717/peerj.6746 (PMC6510221; doi:10.7717/peerj.6746)
Supplement: Tablel S1 [file peerj-07-6746-s001.docx]

| plots | sites | Elevation  (m) | Longitude  (N) | Latitude  (E) | TN  (%) | TC  (%) | TS  (%) | pH | SOC  (mg·g^-1^) | Ammonium  nitrogen  (mg·kg^-1^) | Nitrate nitrogen  (mg·kg^-1^) | Nitrite  nitrogen  (mg·kg^-1^) |
| --- | --- | --- | --- | --- | --- | --- | --- | --- | --- | --- | --- | --- |
| LY1 | LY | 2297 | 38°53'367" | 111°56'499" | 0.29 | 3.42 | 0.05 | 5.89 | 31.40 | 20.84 | 2.82 | 0.18 |
| LY2 | LY | 1950 | 38°52'26" | 112°0'1" | 0.39 | 5.44 | 0.06 | 5.94 | 26.96 | 40.72 | 0.68 | 0.18 |
| LY3 | LY | 2692 | 38°43'349" | 111°50'092" | 0.61 | 9.29 | 0.09 | 5.94 | 40.51 | 40.32 | 0.24 | 0.12 |
| LY4 | LY | 2554 | 38°48'748" | 111°52'077" | 0.41 | 4.89 | 0.06 | 5.96 | 35.18 | 49.64 | 0.42 | 0.14 |
| LY5 | LY | 2432 | 38°43'746" | 111°52'925" | 0.26 | 4.06 | 0.05 | 5.96 | 28.45 | 33.04 | 0.46 | 0.18 |
| PQG1 | PQG | 2649 | 37°53'15.36" | 111°32'44.10" | 0.4 | 5.17 | 0.21 | 6.26 | 28.10 | 11.84 | 3.24 | 0.14 |
| PQG10 | PQG | 1982 | 37°52'54.4" | 111°30'326" | 0.23 | 3.36 | 0.05 | 6.67 | 34.58 | 22.16 | 3.24 | 0.04 |
| PQG2 | PQG | 2652 | 37°53'8.27" | 111°32'45.84" | 0.44 | 6.03 | 0.12 | 6.41 | 29.42 | 9.44 | 3.32 | 0.18 |
| PQG3 | PQG | 2570 | 37°53'5.66" | 111°32'46.02" | 0.52 | 8.68 | 0.11 | 6.04 | 33.46 | 12.08 | 3.16 | 0.18 |
| PQG4 | PQG | 2685 | 37°53'2.58" | 111°32'7.65" | 0.45 | 5.43 | 0.09 | 6.21 | 26.89 | 8.24 | 3.86 | 0.18 |
| PQG5 | PQG | 2671 | 37°52'57.91" | 111°32'7.99" | 0.38 | 4.951 | 0.08 | 6.39 | 24.68 | 12.2 | 3.88 | 0.14 |
| PQG6 | PQG | 2613 | 37°52'55.88" | 111°32'11" | 0.42 | 6.1 | 0.08 | 6.15 | 32.72 | 39.08 | 3.78 | 0.08 |
| PQG7 | PQG | 2400 | 37°53'534" | 111°31'06" | 0.31 | 4.78 | 0.05 | 6.82 | 40.48 | 24.68 | 3.48 | 0.04 |
| PQG8 | PQG | 2227 | 37°53'489" | 111°30'77" | 0.36 | 4.71 | 0.05 | 6.11 | 23.2 | 35 | 3.34 | 0.04 |
| PQG9 | PQG | 2135 | 37°53'164" | 111°30'79" | 0.23 | 3.176 | 0.08 | 7.23 | 43.58 | 12.28 | 1.96 | 0.001 |
| WT1 | WT | 3055 | 39°4'83" | 113°34'094" | 0.62 | 7.56 | 0.09 | 5.81 | 67.12 | 23.68 | 5.14 | 0.06 |
| WT2 | WT | 3040 | 39°4'881" | 113°34'212" | 0.54 | 6.43 | 0.08 | 5.93 | 73.19 | 25.32 | 4.8 | 0.04 |
| WT3 | WT | 3030 | 39°4'877" | 113°34'116" | 0.47 | 5.53 | 0.07 | 6.23 | 72.92 | 26.64 | 4.62 | 0.1 |
| WT4 | WT | 2976 | 39°4'888" | 113°34'12" | 0.60 | 7.01 | 0.12 | 5.87 | 64.53 | 11.48 | 6.76 | 0001 |
| WT5 | WT | 2900 | 39°4'891" | 113°34'215" | 0.56 | 7.28 | 0.08 | 5.56 | 81.89 | 9.6 | 6.78 | 0.04 |
| WT6 | WT | 2891 | 39°4'891" | 113°34'341" | 0.47 | 5.4 | 0.07 | 6.19 | 75.76 | 11.16 | 6.66 | 0.04 |
| WT7 | WT | 2500 | 39°6'33" | 113°56'35" | 0.47 | 5.43 | 0.07 | 6.67 | 65.96 | 15.72 | 8.52 | 0.02 |
| WT8 | WT | 1900 | 39°5'66" | 113.57'26" | 0.37 | 6.6 | 0.05 | 6.68 | 60.93 | 15.68 | 8.34 | 0.02 |
