# Supplementary material for: Deterministic processes dominate soil microbial community assembly in subalpine coniferous forests on the Loess Plateau
Source: PeerJ. 2019 May 7;7:e6746. doi: 10.7717/peerj.6746 (PMC6510221; doi:10.7717/peerj.6746)
Supplement: Table S2 — The detailed information of the sequencing results. [file peerj-07-6746-s002.docx]

| Sample\Info | Seq_num | Base_num | Mean_length | Min_length | Max_length |
| --- | --- | --- | --- | --- | --- |
| PQG7 | 62301 | 28860824 | 463.2482 | 170 | 488 |
| WT1 | 85562 | 38922317 | 454.9019 | 78 | 498 |
| PQG10 | 39766 | 18353471 | 461.5368 | 169 | 488 |
| WT4 | 109188 | 50871482 | 465.9073 | 139 | 488 |
| WT5 | 73202 | 34106827 | 465.9275 | 209 | 490 |
| PQG3 | 18173 | 8427126 | 463.7168 | 96 | 488 |
| PQG1 | 19210 | 8932014 | 464.9669 | 83 | 488 |
| PQG8 | 48292 | 22398689 | 463.8178 | 113 | 490 |
| PQG6 | 18935 | 8760459 | 462.6596 | 230 | 488 |
| PQG2 | 18288 | 8478904 | 463.6321 | 215 | 488 |
| WT3 | 56659 | 26374987 | 465.5039 | 92 | 490 |
| PQG9 | 35454 | 16398422 | 462.5267 | 124 | 489 |
| WT6 | 52680 | 24518599 | 465.4252 | 78 | 587 |
| WT2 | 68118 | 31808295 | 466.9587 | 181 | 494 |
| PQG5 | 16897 | 7837648 | 463.8485 | 189 | 488 |
| WT7 | 93790 | 43734915 | 466.3068 | 88 | 589 |
| WT8 | 58684 | 27308870 | 465.3546 | 96 | 580 |
| LY2 | 34738 | 15211673 | 437.8972 | 317 | 513 |
| LY3 | 32148 | 14051359 | 437.0835 | 294 | 470 |
| LY1 | 31670 | 13893418 | 438.6933 | 299 | 482 |
| LY4 | 34669 | 15232158 | 439.3596 | 292 | 494 |
| LY5 | 39665 | 17329000 | 436.8839 | 279 | 547 |
| PQG4 | 14152 | 6541634 | 462.241 | 168 | 488 |
