# Supplementary material for: Deterministic processes dominate soil microbial community assembly in subalpine coniferous forests on the Loess Plateau
Source: PeerJ. 2019 May 7;7:e6746. doi: 10.7717/peerj.6746 (PMC6510221; doi:10.7717/peerj.6746)
Supplement: Table S3 — The detailed information of molecular ecological networks. [file peerj-07-6746-s003.docx]

| Topological properties | LY | WT | PQG |
| --- | --- | --- | --- |
| Positive correlation | 13 | 65 | 87 |
| Negative correlation | 4 | 0 | 0 |
| Edges | 17 | 65 | 87 |
| Vertices | 20 | 41 | 40 |
| module | 7 | 7 | 6 |
| Connectance | 0.089 | 0.072 | 0.112 |
| Modularity | 0.381 | 0.525 | 0.691 |
| Average degree | 1.711 | 3.171 | 4.35 |
| Average path length | 1.191 | 5.855 | 2.211 |
| Clustering coefficient | 0.667 | 0.452 | 0.858 |
| Betweenness centralization | 0.004 | 0.368 | 0.053 |
| Degree centralization | 0.068 | 0.071 | 0.171 |
| Similarity threshold | 0.6 | 0.6 | 0.6 |
