# Supplementary figures and images for: Deterministic processes dominate soil microbial community assembly in subalpine coniferous forests on the Loess Plateau
Source: PeerJ. 2019 May 7;7:e6746. doi: 10.7717/peerj.6746 (PMC6510221; doi:10.7717/peerj.6746)

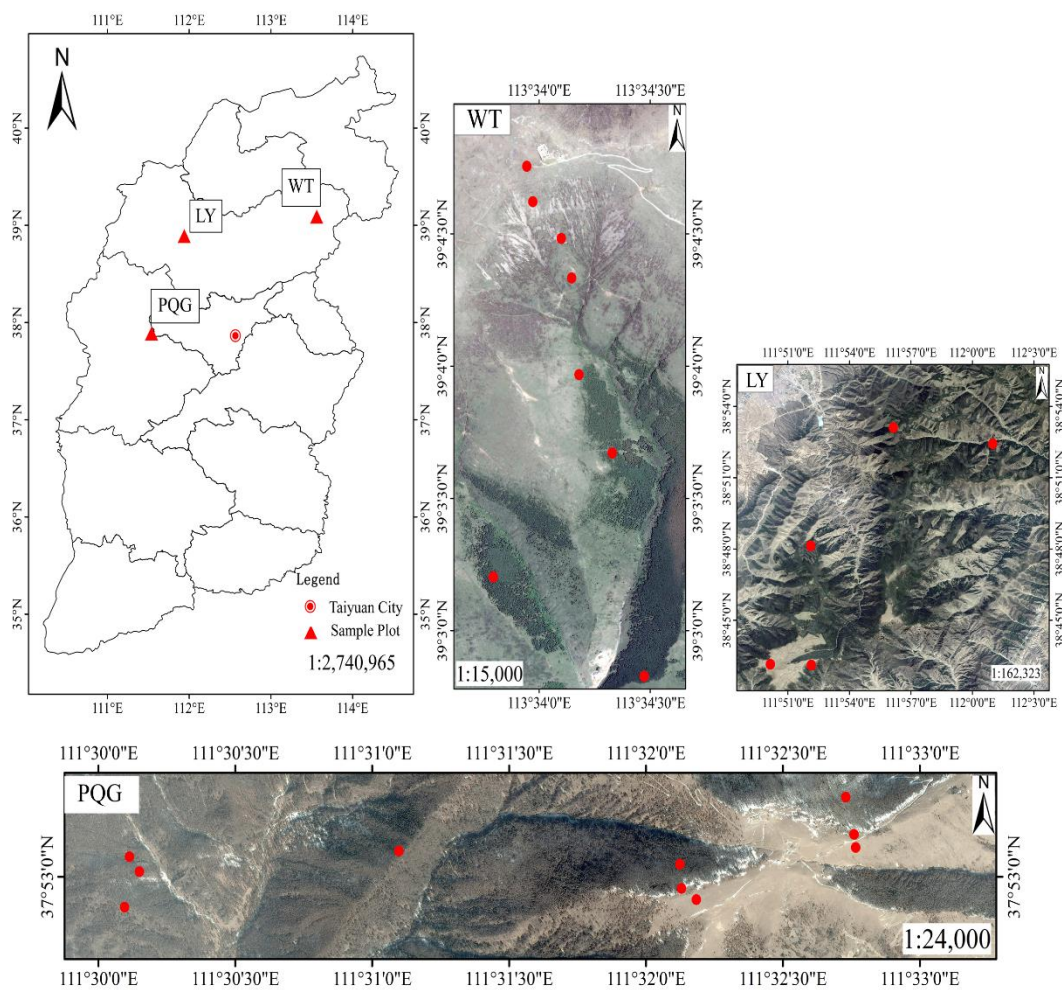

Supplement: Figure S1 [file peerj-07-6746-s004.pdf]
