# Supplementary material for: Deterministic processes dominate soil microbial community assembly in subalpine coniferous forests on the Loess Plateau
Source: PeerJ. 2019 May 7;7:e6746. doi: 10.7717/peerj.6746 (PMC6510221; doi:10.7717/peerj.6746)
Supplement: Dataset S1 — Raw data provided the analytical method (R codes) and the raw data used in this study. [file peerj-07-6746-s005.zip › raw data/null model.zip!/OIK-02803/Appendix_oik.02803.docx]

Oikos	Appendix oik.02803

Tucker, C. M., Shoemaker, L. G., Davies, K. F., Nemergut, D. and Melbourne, B. A. 2015. Differentiating between niche and neutral assembly in metacommunities using null models of β-diversity. – Oikos doi: 10.1111/oik.02803

Appendix 1

Table A1. β-null deviation values for 50 simulations of niche- and neutral-structured metacommunities (types 1 and 5, respectively) for deterministic (A) and stochastic (B) metacommunity models. Values are presented for the presence/absence and abundance versions of the β-null deviation metric (see Methods). Shown are the mean and standard deviations.

| β-null deviation values |  |  |  |  |  |  | |
| --- | --- | --- | --- | --- | --- | --- | --- |
|  | μ | σ |  |  | **μ** | **σ** |  |
| (A) Deterministic |  |  |  | (B) Stochastic |  |  |  |
| Niche |  |  |  | Niche |  |  |  |
| Presence/absence | 0.041 | 0.006 |  | Presence/absence | 0.016 | 0.019 |  |
| Abundance | 0.793 | 0.0001 |  | Abundance | 0.739 | 0.018 |  |
|  |  |  |  |  |  |  |  |
| Neutral |  |  |  | Neutral |  |  |  |
| Presence/absence | 0.000 | 0.000 |  | Presence/absence | –0.027 | 0.062 |  |
| Abundance | –0.341 | 0.000 |  | Abundance | 0.367 | 0.064 |  |

Effect of varying dispersal strength (*m*)

Figure A1. Comparison of the effect of dispersal rate (*m*) on β-null deviation values from simulated metacommunity models, for five assembly types representing a gradient from niche to neutral. Subplots show β-diversity (Jaccard), presence/absence β-null deviation values, and abundance β-null deviation values for stochastic metacommunity models. Results represent 50 replicate metacommunities. Note that y-axes differ for presence/absence and abundance β-null deviation results.

Local dispersal results.
Figure A2. Null deviation results from simulations of deterministic and stochastic metacommunity models with local dispersal for five community types. β-diversity presence/absence, and abundance β-null deviation values for A) deterministic metacommunity models and B) stochastic metacommunity models. Results for both metacommunity types are shown for each of metacommunity types 1–5, and represent 50 replicates (see Methods).
